# Les carcinomes epidermoïdes du scrotum: à propos de 7 cas avec revue de la litterature

**DOI:** 10.11604/pamj.2015.20.163.5991

**Published:** 2015-02-23

**Authors:** Ayoub Halfya, Khalid Elmortaji, Rabii Redouane, Meziane fethi, Amine Rafik, Ezzoubi Mohamed, Chlihi Abdessamad

**Affiliations:** 1 Service d'Urologie du Chu Ibn Rochd, Casablanca, Maroc; 2 Centre Nationale des Brules et de Chirurgie Plastique -CHU Ibn-Rochd, Casablanca, Maroc

**Keywords:** Carcinome épidermoïde, tumeurs génitales, tumeurs cutanées, scrotum, Squamous cell carcinomas, genital tumors, skin tumors, scrotum

## Abstract

Quoique rare le carcinome épidermoïde du scrotum a un mauvais pronostic. Les Carcinomes du scrotum induite et - liées au travail sont moins fréquentes en raison d'une meilleure hygiène, vêtements de protection, et la sensibilisation de la cancérogénicité des huiles industrielles. L’épidémie à l'HPV a induit une augmentation de l'incidence. Le traitement de dépend toujours exérèse locale de la lésion primaire. La radiothérapie a peu de bénéfice thérapeutique dans le traitement d'un carcinome épidermoïde du scrotum. La bléomycine peut être utile comme traitement adjuvant pour les maladies ilio-inguinal généralisée avant la tentative exérèse, même si cela n'a pas encore été prouvé. Entre janvier 2011 au 1^er^ janvier 2013, 7 patients atteints de carcinome épidermoïde ont été pris en charge, Trois patients ont présenté une localisation ganglionnaire. Les sept patients ont eu un traitement chirurgical par exérèse large avec reconstruction, Deux patients ont été adressé pour chimiothérapie.2 patients ont présenté une récidive, dont un est décédé.

## Introduction

L'incidence des carcinomes scrotaux est très rares [1], la série la plus large dans la littérature présente 28 cas [2–5]. La forme histologique la plus fréquente est le carcinome épidermoïde [6]. Elle survient le plus souvent dans les soixantaines sur une lésion préexistante, augmentant progressivement de volume, elle est associée dans 25% des cas à des adénopathies inguinales. La chirurgie d'exérèse éventuellement associée à un curage ganglionnaire en fonction du stade reste le traitement de référence. L'objectif de notre série est de décrire les caractéristiques épidémiologiques, diagnostiques et thérapeutiques.

## Méthodes

Sur une période de deux ans (1^er^ janvier 2011 au 1erjanvier 2013), nous avons réalisés une étude rétrospective sur 7 cas de carcinome épidermoïde colligés au centre nationale des brûlés et de chirurgie plastique du CHU Ibn Rochd Casablanca. Les données épidémiologiques, diagnostiques, et thérapeutiques ont été relevées.

## Résultats

**Figure 1:**
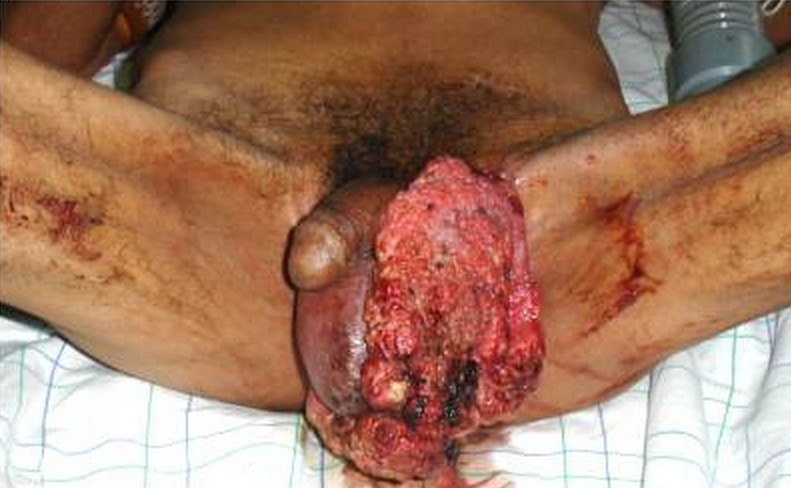
Photo montrant une lésion ulcéro-bourgeonnante bilatérale du scrotum chez un patient atteint de VIH

**Figure 2:**
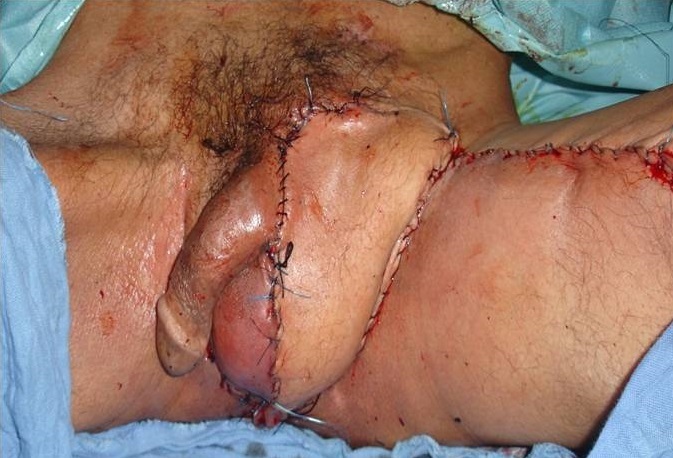
Carcinome épidermoïde bilatérale avec reconstruction par un lambeau fascio-cutané de la cuisse gauche

Les caractéristiques des patients ont été détaillées dans le (Tableau 1). L’âge moyen au diagnostic était de 64 ans (48-80 ans). Le mode de révélation chez tous les patients était la présence d'une lésion macroscopique (Figure 1), la taille médiane de la lésion était 4 cm (1,5-8 cm). Trois patients ont présenté une localisation ganglionnaire (N1 et N2). Les sept patients ont eu un traitement chirurgical au début par exérèse large avec reconstruction immédiate dans 2 cas. Les techniques de reconstruction utilisées sont en fonction de l’étendue de la perte de substance. Ainsi, nous avons eu recours au greffes cutanées dans 2 cas, le lambeau fascio-cutanée de la cuisse dans 2 cas (Figure 2), et lambeaux d'avancement scrotal dans 3 cas. Aucune marge d'exérèse positive n'a été rapportée, deux curage ganglionnaire ont été réalisé et dont un bilatérale qui s'est avéré positif d'un seul côté, avec un ganglion >3 cm, et effraction capsulaire. Deux patients ont été adressé pour chimiothérapie, l'un à visée néo adjuvants à base de (Bléomycine - Méthotréxate-Cisplatine), l'autre dans un objectif palliatif. La médiane de suivi a été de 12 mois (6 -16mois). 2 patients ont présenté une récidive, dont un est décédé, venu d'emblée avec un stade métastatique.

**Table 1:** Les caractéristiques épidémiologiques, diagnostiques et thérapeutiques des patients

Patient	Age	Facteur Prédisposant	clinique	TNM	Taille de la PDS	Technique de reconstruction	récidive
1	80	lichen	Multiples nodules verruqueux bilatérale	T2+ N1+ M0	7,6×4×2cm	Greffe cutanée+ curage inguinale unilatérale	Non
2	70	Partenaires multiples	Lésion Ulcéro-bourgeonnante gauche	T2+ N1+ M0	6×3,4×1,7cm	Lambeau scrotal d'avancement	Non
3	69	Condylome	Lésion Ulcéro-bourgeonnante gauche	T2+ N0+ M0	2×1,8×0,8cm	Lambeau scrotal d'avancement	Non
4	64	Condylome	Lésion Ulcéro-bourgeonnante gauche	T2+ N0+ M0	6,5×5×3,4cm	Lambeau scrotal d'avancement	Non
5	48	Homosexualité	Placard nodulaire et fistuleux bilatérale	T3+ N1+ M0	8,5×5×4,5cm	Lb fascio-cutanée en VY de la face interne des cuisses+ curage inguinale unilatérale	Oui
6	75	Condylome	Nodule droit	T2 N0 M0	2,8×1,5×2cm	Greffe cutanée	Non
7	52	HIV	Lésion Ulcéro-bourgeonnante bilatérale	T3 N2 M1	8,8×6,7×3cm	Lb fascio-cutanée en VY de la face interne des cuisses+ curage inguinale bilatérale	Décédé

## Discussion

Le carcinome du scrotum est une tumeur rare avec une incidence annuelle globale d'environ 1,5 par 1.000.000 personnes dans les pays occidentaux [7]. Wright et al a recueilli 471 cas de cancer du scrotum 1973-2002 et trouvé types histologiques les plus fréquents étaient le carcinome épidermoïde (32%), la maladie de Paget extra-mammaire (21%), carcinome baso-cellulaire (18%) et le sarcome (18%). Le carcinome épidermoïde est plus fréquent chez les hommes de race noire que chez les hommes blancs (69 vs 31%) [8]. Carcinome du scrotum a été le premier cancer lié à l'exposition professionnelle. Des études antérieures ont montré, qu'il a été le plus souvent associé à l'exposition à des carcinogènes environnementaux tels que la suie de cheminée, goudrons, de la paraffine, et certains produits pétroliers [9]. Actuellement la plupart des cas résultent d'une mauvaise hygiène et l'inflammation chronique [10]. Les patients que nous rapportons ici avaient des condylomes dans 42%, des lésions inflammatoires du scrotum dans 28%, ce qui suggère que l'inflammation chronique et la mauvaise hygiène peut contribuer à l'apparition de la maladie. En général, la récidive était précoce chez les patients présentant des caractéristiques histologiques squameuses, par rapport à d'autres sous-types histologiques (Johnson et al. [11]). Dans notre série la forme histologique la fréquente est la forme ulcéro-bourgeonnante dont le la malignité reste principalement local. La modalité de traitement primaire pour le cancer du scrotum est la chirurgie. Dai et al. [12] ont rapporté sur 10 patients atteints de cancer du scrotum dont tous les patients ont été traités par exérèse chirurgicale large sans traitement adjuvant avec un moyen de survie globale de 118 mois. Dans notre étude, après un suivi moyen de 12 mois, 5 patients étaient en bonne santé, sans rechute. Un patient a développé des métastases ganglionnaires gauche à 15 mois, et qui a été traité avec succès par un curage ganglionnaire inguinale associe à la chimiothérapie. Un patient a décédé venu d'emblée avec des métastases pulmonaires bilatérales. Le traitement néoadjuvant (à la fois chimiothérapie et radiothérapie) a également été recommandé pour réduire la taille de la tumeur et les adénopathies, modifiant ainsi le stade tumorale [13, 14]. La Radiothérapie adjudante en combinaison avec la chimiothérapie (méthotrexate, bléomycine et cisplatine) pour quatre cycles est également recommandée afin de parvenir à une meilleure survie sans rechute [15].

## Conclusion

La faible incidence des carcinomes épidermoïdes du scrotum dans la population générale reste un obstacle concret à la publication de séries conséquentes de patients, susceptibles de générer une prise en charge thérapeutique bien codifiée. Le pronostic du carcinome épidermoïde est conditionné par sa profondeur d'infiltration, son grade histo-pronostique et la précocité de la prise en charge. La surveillance des lésions précancéreuses et les mesures d'hygiènes restent le meilleur moyen de prévention.
